# Nucleotide Variation in *Drosophila cryptochrome* Is Linked to Circadian Clock Function: An Association Analysis

**DOI:** 10.3389/fphys.2022.781380

**Published:** 2022-02-17

**Authors:** Mirko Pegoraro, Emily Sayegh Rezek, Bettina Fishman, Eran Tauber

**Affiliations:** ^1^School of Biological and Environmental Sciences, Liverpool John Moores University, Liverpool, United Kingdom; ^2^Department of Genetics and Biology, University of Leicester, Leicester, United Kingdom; ^3^Department of Evolutionary and Environmental Biology, Institute of Evolution, University of Haifa, Haifa, Israel

**Keywords:** association mapping, circadian clock, cryptochrome, *Drosophila*, genetic cline, genetic variation, molecular polymorphism

## Abstract

Cryptochrome (CRY) is a conserved protein associated with the circadian clock in a broad range of organisms, including plants, insects, and mammals. In *Drosophila*, *cry* is a pleiotropic gene that encodes a blue light-dedicated circadian photoreceptor, as well as an electromagnetic field sensor and a geotaxis behavior regulator. We have generated a panel of nearly-isogenic strains that originated from various wild populations and which carry different natural alleles of *cry*. Sequencing of these alleles revealed substantial polymorphism, the functional role of which was elusive. To link this natural molecular diversity to gene function, we relied on association mapping. Such analysis revealed two major haplogroups consisting of six linked nucleotides associated with circadian phase (haplotypes All1/All2). We also generated a maximum-likelihood gene-tree that uncovered an additional pair of haplogroups (B1/B2). Behavioral analysis of the different haplotypes indicated significant effect on circadian phase and period, as well on the amount of activity and sleep. The data also suggested substantial epistasis between the All and B haplogroups. Intriguingly, circadian photosensitivity, assessed by light-pulse experiments, did not differ between the genotypes. Using CRISPR-mediated transgenic flies, we verified the effect of B1/B2 polymorphism on circadian phase. The transgenic flies also exhibited substantially different levels of *cry* transcription. We, moreover, analyzed the geographical distribution of the B1/B2 haplotypes, focusing on a 12 bp insertion/deletion polymorphism that differentiates the two haplotypes. Analysis of *cry* sequences in wild populations across Europe revealed a geographical cline of B1/B2 indel frequency, which correlated with seasonal bioclimatic variables. This spatial distribution of *cry* polymorphism reinforces the functional importance of these haplotypes in the circadian system and local adaptation.

## Introduction

The circadian clock system is one of the best examples of a molecular circuit that can generate an organized output without any external cues, yet which is amenable to environmental modulators, such as temperature and light. Entrainment of the pacemaker to daily temperature or light–dark cycles adjusts the period to 24 h and sets the system to a phase, which is ecologically adaptive (Johnson et al., 2003). Given the broad range of temperature and light conditions to which wild populations are exposed, it is not surprising that molecular adaptations in circadian clock genes are pervasive and accordingly, have been reported in various phyla, such as plants (Endo, 2016), insects (Numata et al., 2015), and vertebrates (Johnsen et al., 2007). Furthermore, temperature and light vary systematically with latitude and consequently may lead to latitudinal clines in circadian clock phenotypes and in molecular polymorphisms in clock genes (Kyriacou et al., 2008; Hut et al., 2013). The threonine-glycine (TG) repeat polymorphism within the *Drosophila period* (*per*) gene was the first such cline to be described (Costa et al., 1992). In northern Europe, the frequency of the two major alleles, TG17 and TG20, follows a cline. Specifically, TG20 is more abundant at high latitudes, whereas TG17 is more common in the south. The possibility of a similar cline in Australia has been debated (Weeks et al., 2006; Kyriacou et al., 2007). Laboratory experiments showed that TG polymorphism is associated with thermal adaptation (Sawyer et al., 1997). Flies carrying the TG17 allele have a free-running period (FRP) very close to 24 h at high temperatures and are, therefore, well adapted to the warmer regions of Europe. Flies with the TG20 variant show superior temperature-compensation, which fits well with the higher thermal variability seen in the northern latitudes.

A polymorphism in the *timeless* (*tim*) gene also follows a latitudinal cline (Tauber et al., 2007). This polymorphism consists of a single base insertion/deletion in the 5′ UTR region located between two translation start codons. The allele with the deletion yields only the short isoform (from the downstream ATG) and is called *s-tim*, whereas the allele with the insertion is translated to both the long and short isoforms (*ls-tim*). Rather than affecting thermal adaptation, the *tim* polymorphism has been shown to impact circadian photosensitivity and photoperiodism (Sandrelli et al., 2007). The frequency of *s-tim* in European populations is higher in northern regions, whereas *ls-tim* prevails in southern regions. However, analysis of the variation pattern suggested that cline can explain the fact that *ls-tim* is the derived allele that has recently emerged in southern Italy and is currently spreading across Europe by directional selection (Tauber et al., 2007; Zonato et al., 2018). Interestingly, the study of *tim* polymorphism was vital for the discovery of another protein named JETLAG, an F-box protein, which binds to TIM and induces TIM ubiquitinization (Koh et al., 2006). The phenotype of JETLAG mutants, which are rhythmic in continuous light, is expressed only in strains carrying the *ls-tim* allele and not in those carrying the *s-tim* allele (Peschel et al., 2006). These discoveries demonstrate how understanding natural genetic variation in clock genes can help explain the evolution and molecular mechanisms that drive the clock.

Cryptochrome (CRY) is an evolutionary conserved blue-light photoreceptor associated with light entrainment of the circadian clock in *Drosophila* (Emery et al., 1998; Thompson and Sancar, 2002). Research in recent years suggested that *cry* is a pleiotropic gene involved in multiple cellular processes. Light-activated CRY targets clock proteins for proteasomal degradation (Naidoo et al., 1999; Lin et al., 2001; Busza et al., 2004). It also drives membrane depolarization in clock neurons (l-LNvs) through modulation of potassium ion channels (Fogle et al., 2011). Additionally, CRY is involved in the response of *Drosophila* to low-intensity electromagnetic fields (Gegear et al., 2008; Yoshii et al., 2009; Fedele et al., 2014) and in the negative geotaxis exhibited by adult flies (Toma et al., 2002; Fedele et al., 2014).

We previously carried out a survey of natural allelic variation in *cry* (Pegoraro et al., 2014). The screen, based on a small number of coding DNA sequences, focused on the widespread missense single-nucleotide polymorphism (SNP) L232H. The intermediate frequencies of the two alleles across many wild populations suggested that the variation observed was driven by balancing selection. The pattern of variation associated with this SNP and the high level of linkage disequilibrium in the *cry* locus supported this interpretation. We further identified circadian phenotypes associated with the L232H SNP, namely, those in which the phase of locomotor activity and adult eclosion were affected.

In the present study, we generated a panel of 33 near-isogenic lines (NIL) carrying *cry* alleles that were introgressed from various wild population strains. These lines were generated by backcrossing wild strains to deficiency line for eight generations and therefore theoretically share 99.79% of their genomes, excluding the *cry* locus (although the possibility remains that residual genetic variability in other loci also contributes to the phenotypes of interest). We used these lines for fine association mapping of polymorphic sites in the gene with circadian clock properties.

## Materials and Methods

### Fly Strains

To generate *Drosophila melanogaster* isogenic lines with natural *cry* alleles, (P) single males were isolated from 33 isofemales lines (Supplementary Table S1) and crossed with deficiency [Df(3R)Dl-BX12, ss^1^ e^4^ ro^1^/TM6B, Tb^1^; stock number 3012, cytological position 91F1-2; 92D3-6] virgin females. The deficiency overlaps with the *cry* gene (cytological position 91F11). A single non-*Tb* virgin female (F1) was then isolated and crossed with Df males. This back-cross was repeated for eight generations (F1–F8). To remove the Df from the isogenic line, *Tb* males and females (F9) were crossed, and non-tubby pupae were isolated in the following generation (F10). Flies were maintained for the entire experiment in a 12 h light/12 h dark cycle at 25°C (LD 12:12). Lines were maintained in plastic vials (10 × 2 cm) in standard cornmeal food.

### *Cry* Allele Sequencing

DNA was isolated from 5 to 10 flies per line using 300 μl of extraction buffer (0.25 mM EDTA, pH 8, 25 mM NaCl, 10 mM Tris–HCl, pH 8) supplemented with proteinase K to a final concentration of 0.2 mg/ml. The flies were homogenized and incubated at 37°C for 45 min. Proteinase K was deactivated by incubation at 95°C for 5 min. The isolated DNA was either used immediately or stored at −20°C. The *cry* genomic region was PCR-amplified (35 cycles: 92°C for 30 s, 58°C–62°C for 30 s, 72°C for 2 min and 30 s) using a combination of different primers (Supplementary Table S2). The reaction mix contained the primers (0.5 μM), 4.5 μl of PCR buffer (dNTPs (1 mM each), 11 mM ammonium sulfate, 4.5 mM MgCl_2_, 6.7 mM 2-mercaptoethanol, 4.4 mM EDTA, pH 8, 113 μg/ml BSA, 45 mM Tris–HCl, pH 8), 1.5 μl Taq-pfu mix (M7841 and M7741, Promega) and 2 μl of DNA in a final volume of 50 μl. The amplification was separated in a 1% agarose gel and the *cry* amplicon was gel purified using a Zymoclean Gel recovery kit (D4002) following the manufacturer’s instructions. The purified bands were Sanger-sequenced (Beckman Coulter Genomics) using the primers listed in Supplementary Table S2. All sequences were deposited at GenBank under accession numbers MW758991–MW759024.

### Polymorphism Analysis

DnaSP (Ver5.10.01) was used to analyze *cry* genetic variation (Librado and Rozas, 2009). Three lines (HOJ12_133, REN12.1, and HOJ34_111) where excluded from the polymorphism analysis because their sequence was not complete. For the McDonald Kreitman test (McDonald and Kreitman, 1991) and maximum likelihood phylogenetic analysis, *cry* genomic sequences of *Drosophila simulans* were included (Hu et al., 2013; Lack et al., 2016).

### Locomotor Activity and Circadian Behavior

Locomotor activity was recorded as previously described (Rosato and Kyriacou, 2006) at 25°C. Using the TriKinetics system, we recorded the locomotor activity of 3–4 day-old males maintained initially for 3–5 days in LD 12:12, followed by 5 days in constant dark (DD) at 25°C. The flies were then re-entrained for 4 days in LD 12:12. A 20 min light pulse (saturating white light >500 lux) was given at Zeitgeber Time 15 (ZT15; 3 h after lights-off) during the last night, before releasing the flies to DD for a further 5 days.

The phase of activity in LD 12:12 was analyzed on the third day of LD 12:12. Given the bimodal locomotor activity profiles of *Drosophila*, phase was determined for both the morning and evening activity peaks. Circular statistic software (Oriana, Kovach Computing Services, United Kingdom) and the Watson-Williams *F*-test were used to analyze the phase data. To test whether population mean ranks differed, we conducted the Wilcoxon Signed-rank test and a one-way permutation test based on 9,999 Monte Carlo simulations (Wilcoxon, 1945).

The amount of sleep, defined as 5 min of inactivity or longer (Hendricks et al., 2000; Tononi, 2000), and total amount of activity per day were also recorded during the LD 12:12. Sleep amount in LD 12:12 was analyzed separately during the 12 h of light (min/12 h) and the 12 h of dark (min/12 h) using nested ANOVA. We re-sampled across all groups to calculate 95% confidential limits for *F* scores.

The activity data collected for the first 5 days in DD were used to calculate the free-FRP and acrophase (*φ*) using cosinor-rhythmometry (Nelson et al., 1979). Changes in the FRP were analyzed using nested ANOVA and re-sampling to calculate the *F* score 95% confidential limit.

The difference between the phase on the third day after the light pulse (φlp) and the reference phase (φref: phase of DD before the light pulse) was calculated with a custom-made Excel macro. Circular statistic software (Oriana) and a Watson-Williams *F*-test were used to analyze the phase data.

### Genotype–Phenotype Association

To estimate associations between phenotypic traits and genotypes, we used TASSEL software (Trait Analysis by aSSociation, Evolution and Linkage; Bradbury et al., 2007). From the full alignment of 33 wild-type *cry* sequences, a file containing only the most common 53 SNPs and indels (present in 10 or more lines, cut-off 30%) was generated. This file was used to test associations between the different circadian phenotypes and genetic variation in *cry*. The phenotypes were represented by average values per line. The association was tested using a General Linear Model, a function that uses a fixed effects regression model. A main effect-only model was automatically built using all variables in the input data. Each trait by marker combination was tested and trait by marker *F*-tests were produced. The algorithm runs a permutation test which calculates the predicted and residual values of the reduced model, permutes the residuals (1,000 permutations) and adds them to the predicted values (Anderson et al., 2003).

### CRISPR-Mediated Homology-Directed Repair

We used CRISPR-mediated homology-directed repair (HDR) to insert All2 or B1 alleles into the same genetic background (*act-cas9: y1* P(*act5c-cas9, w*+) M(3xP3-RFP.attP)ZH-2A w*; Bloomington 24,480), as previously described (Gratz et al., 2013). To drive the HDR, two sgRNAs for the All haplotypes and two for the B haplotypes were cloned into plasmid pCFD4 using the primers listed in Supplementary Table S2 and a Gibson Assembly kit (E5510 NEB). The sgRNAs complemented the PAM site at positions 1,744–1,746 (5′All) and 2,108–2,110 (3′All) and 2,850–2,852 (5′B) and 3,238–2,852 (3′B). To promote HDR, the *act-cas9* line used was All1B2 and single-stranded oligonucleotides (ssODNs) encoding All2 and B1 (both synthesized by Genescript, Hong Kong) were employed. Each ssODN included 60 nucleotide homology arms left of the 5′ PAM site and right to the 3′ PAM site. In the ssODNs design, the sequence of the All2 and B1 haplotypes was the most common nucleotide per significant site per haplotype. For the not informative site, the *act-cas9* sequence was maintained. Injection of the sgRNA pCDF4-expressing vectors and ssODH was done using the microinjection service of the Department of Genetics, University of Cambridge. This procedure yielded two recombinant lines (All2B2 and All1B1).

### RNA Extraction and qPCR Analysis

Transgenic (All1B1, All2B2) and *act-cas 9* (All1B2) flies were collected in liquid nitrogen at ZT01 and ZT13 (1 h after light on and light off respectively). About 50 heads from each sample were used for total RNA extraction using the PureLink RNA Mini kit (Invitrogen). First-strand cDNA was synthetized using 500 ng RNA of each sample, with the High-Capacity cDNA Reverse Transcription kit (Applied Biosystems). The cDNA samples were diluted (10×), and 1 μl was used in quantitative PCR (qPCR) (FastSYBR™ Green Master Mix, Thermo Fisher). The primer sequences are listed in Supplementary Table S2. The *Rpl32* gene served as a reference for gene expression. We used the QuantStudio™ 3 Real-Time PCR System (Applied Biosystem). A standard curve was plotted for each of the genes, and four biological replicates were analyzed for each condition. The cycle crossing point (Cp) of each reaction was calculated by QuantStudio software version 1.5.

## Results

### Genetic Variation

Analysis of the *cry* allele sequences in 33 NIL strains revealed substantial genetic variation, including 32 synonymous and 16 non-synonymous changes (Supplementary Table S3). Nucleotide diversity was estimated using *θ*s to be 0.011 ± 0.0034 (SD) and *π* to be 0.0104 ± 0.00065.

**Figure 1:**
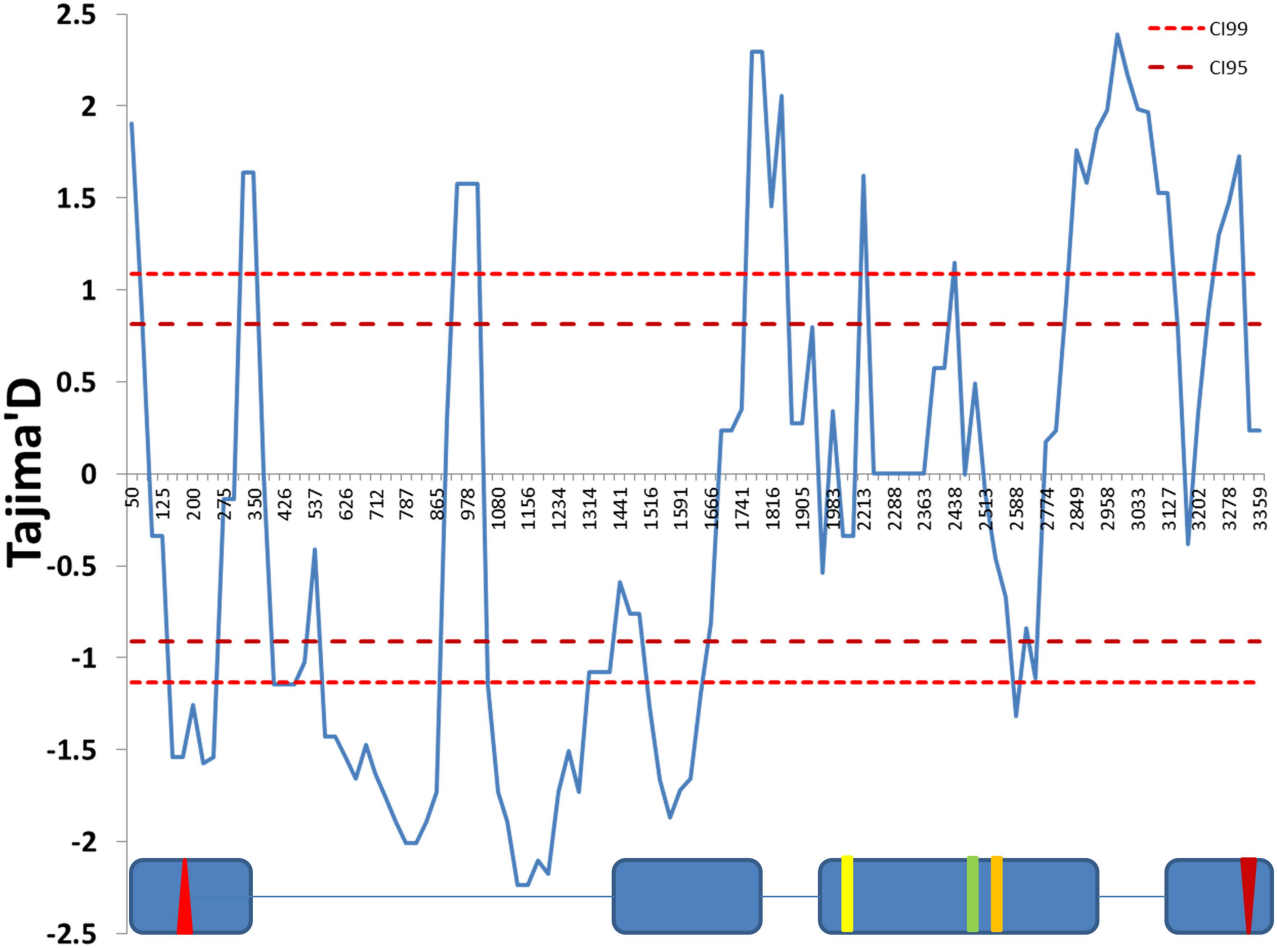
Sliding window analysis of Tajima’s D neutrality test statistics. The figure shows a sliding window of Tajima’s D calculated every 100 bp, with a window size of 25 bp. The 99% and 95% CIs were produced by coalescent simulation using *θ* = 31.04, *n* = 30, number of replications = 1,000 and estimated recombination per gene *R* = 41.199. The exon (blue boxes) and intron (lines) structure of the *cry* genomic region is depicted below. The red up- and down-oriented triangles indicate the start and stop codons, respectively. Yellow, green, and orange vertical lines indicate three non-synonymous substitutions (L232H, E335D, and D348N, respectively). Nucleotide positions are numbered beginning from 10 nucleotides before the first exon, relative to the *cry* sequence (FBgn0025680).

We used Tajima’s D statistics (Tajima, 1989) to test for the signature of selection at this locus. A sliding window analysis revealed both negative and positive significant peaks (Figure 1). The first part of the sequence, in particular the first intron, showed significant negative values of Tajima’s D, while significant positive peaks were present at the end of the sequence, suggesting that different selection scenarios act across the gene.

To test for adaptive evolution, we used the McDonald-Kreitman test (McDonald and Kreitman, 1991). As this test compares the ratio of non-synonymous to synonymous variation within and between different species, we compared *cry* sequences from four *D. simulans* strains. We found 127 fixed and 215 polymorphic silent changes (Ds, Ps) and one fixed and 20 polymorphic (Dn, Pn) replacement changes. The neutrality index was significantly higher than unity (Ni = 11.81, Fisher’s exact test *p* = 0.002), indicating departure from neutrality due to an excess of non-silent polymorphism.

**Figure 2:**
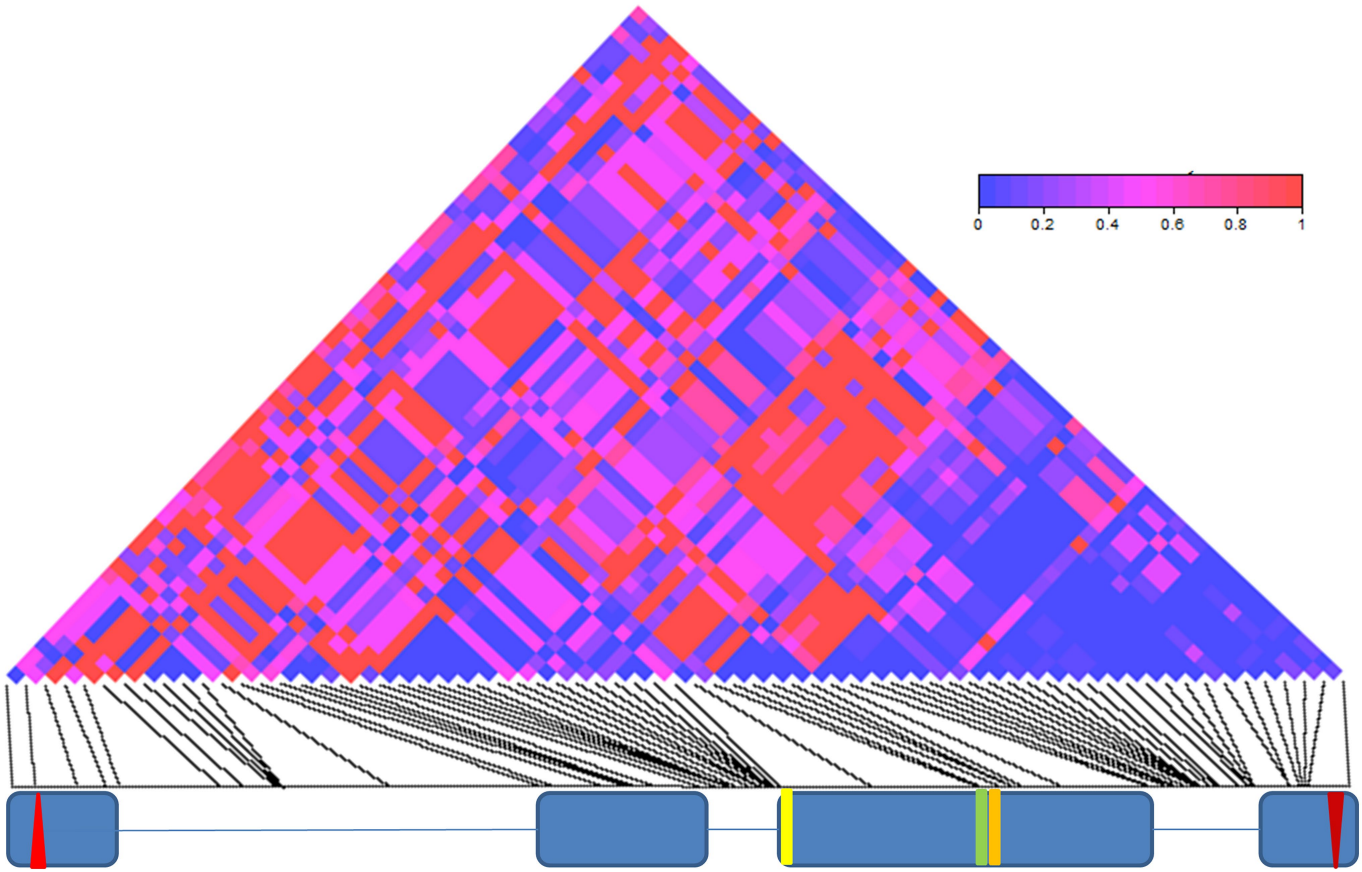
Linkage disequilibrium (LD) in *cry*. LD across the 3,848 bp-long *cry* genomic region is depicted as a matrix of pairwise comparisons among 69 informative sites. Values of *p* (Fisher’s exact test) are shaded according to level of significance (see color code). The exon (blue boxes) and intron (lines) structure of *cry* is depicted below. The red up- and down-oriented triangles indicate the start and stop codons, respectively. Yellow, green, and orange vertical lines indicate three non-synonymous substitutions (L232H, E335D, and D348N, respectively).

We also have found an excess of linkage-disequilibrium between the 69 informative sites (70 significant pairwise comparisons, after Bonferroni correction; Supplementary Table S4; Figure 2). The significant linkage-disequilibrium loci were organized in four main blocks, a group of 10 sites within intron II, a small cluster of four sites around two missense mutations in exon III (E335D and D348N), a large cluster of 18 sites within intron III and a small group of three sites within exon IV, within 150 bp of the stop codon (Supplementary Table S4; Figure 2).

**Figure 3:**
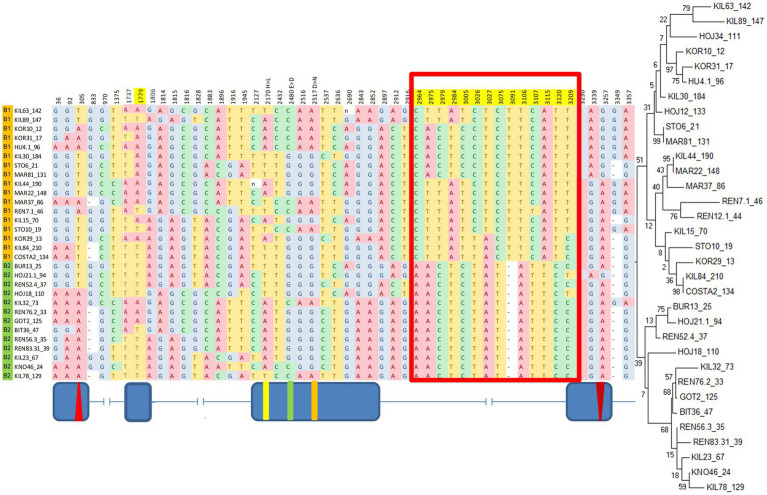
B1/B2 haplogroups revealed by a maximum-likelihood gene tree of *cry*. Red boxes indicate the polymorphism underlying the two clusters in the phylogenetic tree. Fly strains are listed according to their gene tree position. Top branches: haplogroup B1 (orange); Bottom branches: haplogroup B2, (green). Position 3,091 represents a 12 bp indel (truncated for clarity). Shown are polymorphic sites with minor allele frequency >30%. The maximum-likelihood gene tree is shown on the right. Schematic diagram (not to scale) indicating the exon–intron is shown below.

A maximum-likelihood gene tree for *cry* revealed two major clades (Figure 3), which define two broad haplotypes that we named B1 and B2. Nine SNPs and a single indel (12 bp-long) underlie the differences between the clades. These SNPs form an LD block (Supplementary Table S3; Figure 2) in the intron III-beginning of exon IV (from position 2,964 to position 3,209; Figure 3), including the 12 nucleotide-long insertion/deletion (indel) at position 3,091 (Figure 3).

### Phenotypic Analysis

**Figure 4:**
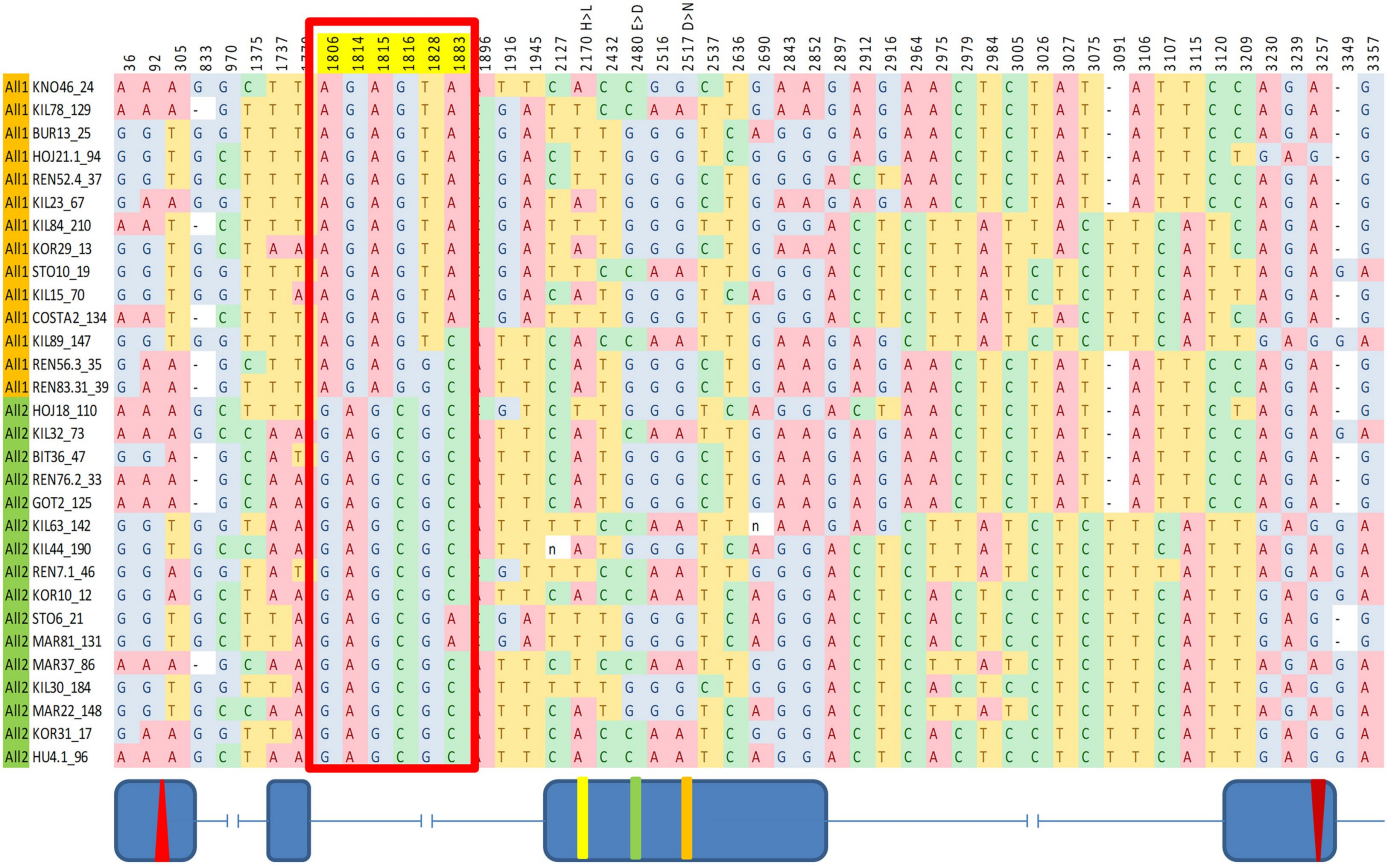
Fine mapping of SNPs in *cry* associated with circadian acrophase. The red box indicates six SNPs showing a significant association. Fly strains are organized accordingly to two haplotype groups (All1, orange; All2, green). These SNPs form a linkage group within intron II. Schematic diagram (not to scale) indicating the exon–intron is shown below.

SNPs across the entire *cry* region were tested for association with various circadian traits. We identified a significant association between a group of six SNPs within intron II and the DD acrophase (Figure 4). These SNPs form a linkage cluster that defines two haplotypes, which we named All1 and All2. These haplotypes are distinct from the B1/B2 haplotypes identified in the maximum-likelihood gene tree. It is noteworthy that the Tajima D analysis indicated significant positive values in both the A and B regions, suggesting that both polymorphisms are driven by balancing selection (Figure 1).

**Figure 5:**
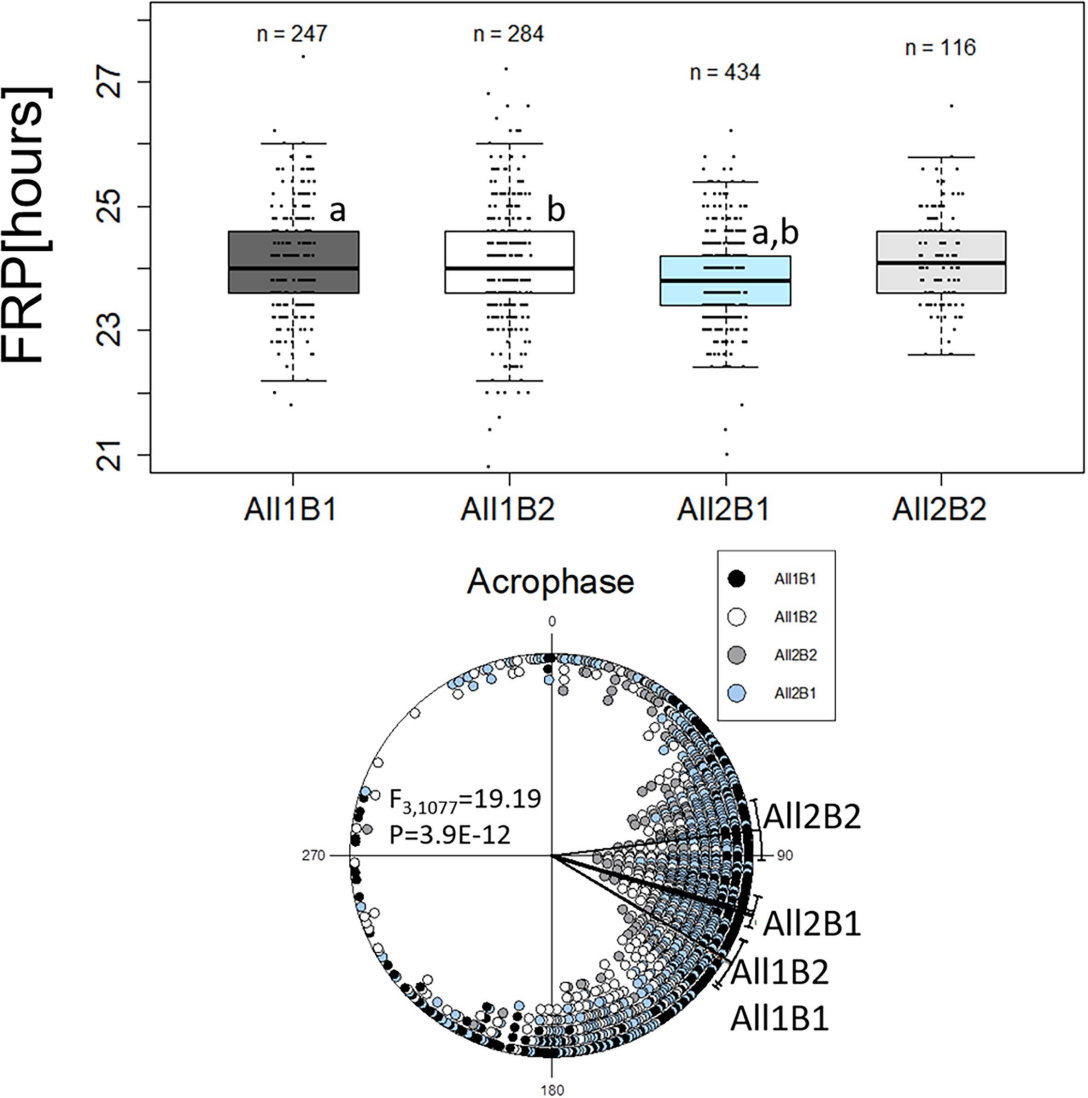
Circadian free-running period (FRP) and acrophase of *cry* haplotypes. The FRP (**upper panel**) is depicted using a boxplot, where the middle line represents the median, and the box indicated the interquartile range (IQR). Whiskers signify 1.5 × IQR. White circles are potential outliers. Similar letters on boxplots indicate significant differences revealed by *post-hoc* tests (a, b, c, *p* < 0.05). FRP: All1B1 24 h ± 0.07 h (median ± SEM), All1B2 24 h ± 0.1 h, All2B1 23.8 h ± 0.03 h and All2B2 24.1 h ± 0.07 h. The circadian acrophase (phase in DD; **lower panel**) was calculated over 5 days and is depicted by circular plots. Each symbol represents a single observation. 0° and 180° correspond to subjective light on (CT0) and light off (CT12), respectively (15° = 1 h). Acrophases: All1B1 121.16° ± 57.56° (mean vector ± circular standard deviation, *n* = 247); All1B2 120.61° ± 56.17° (*n* = 284); All2B1 105.46° ± 46.50° (*n* = 434); and All2B2 82.91° ± 46.21° (*n* = 116).

We re-analyzed the phenotypic data from the NIL, grouping the lines according to both haplotypes All1/All2 and B1/B2. The FRP of All2 flies was shorter than that of All1 flies (*F*_1,1,048_ = 14.33, *p* = 1.42E-04), while the FRP of B1 flies was shorter than that of B2 flies (both at about 0.2 h, *F*_1,1,048_ = 7.75, *p* = 5.46E-03; Figure 5). This difference increased to 0.3 h when comparing the genotypes of both haplotypes (*F*_3,1,048_ = 6.99, *p* = 1.17E-04; Figure 5).

The acrophase of All1 flies was significantly delayed, as compared to that of All2 flies, while there was no significant effect seen with the B haplotypes (*F*_1,1,079_ = 38.91, *p* = 6.35E-10; Figure 5). Comparing the full haplotypes revealed intricate effects (Figure 5). The most advanced acrophase was presented by All2B2 flies that was significantly different from all other haplotypes. Acrophase in All2B2 flies was more advanced than that of All2B1 (*F*_1,548_ = 21.17, *p* = 5.22E-06), All1B2 (*F*_1,398_ = 41.7, *p* = 3.10E-10) and All1B1 flies (*F*_1,361_ = 39.95, *p* = 7.70E-10). Acrophase in All2B1 flies was significantly advanced, relative to that of All1B1 flies (*F*_1,679_ = 14.02, *p* = 1.97E-04) and All1B2 (*F*_1,716_ = 14.64, *p* = 1.42E-04). These results suggest that epistasis between the All and B haplotypes contributed to acrophase variation.

**Figure 6:**
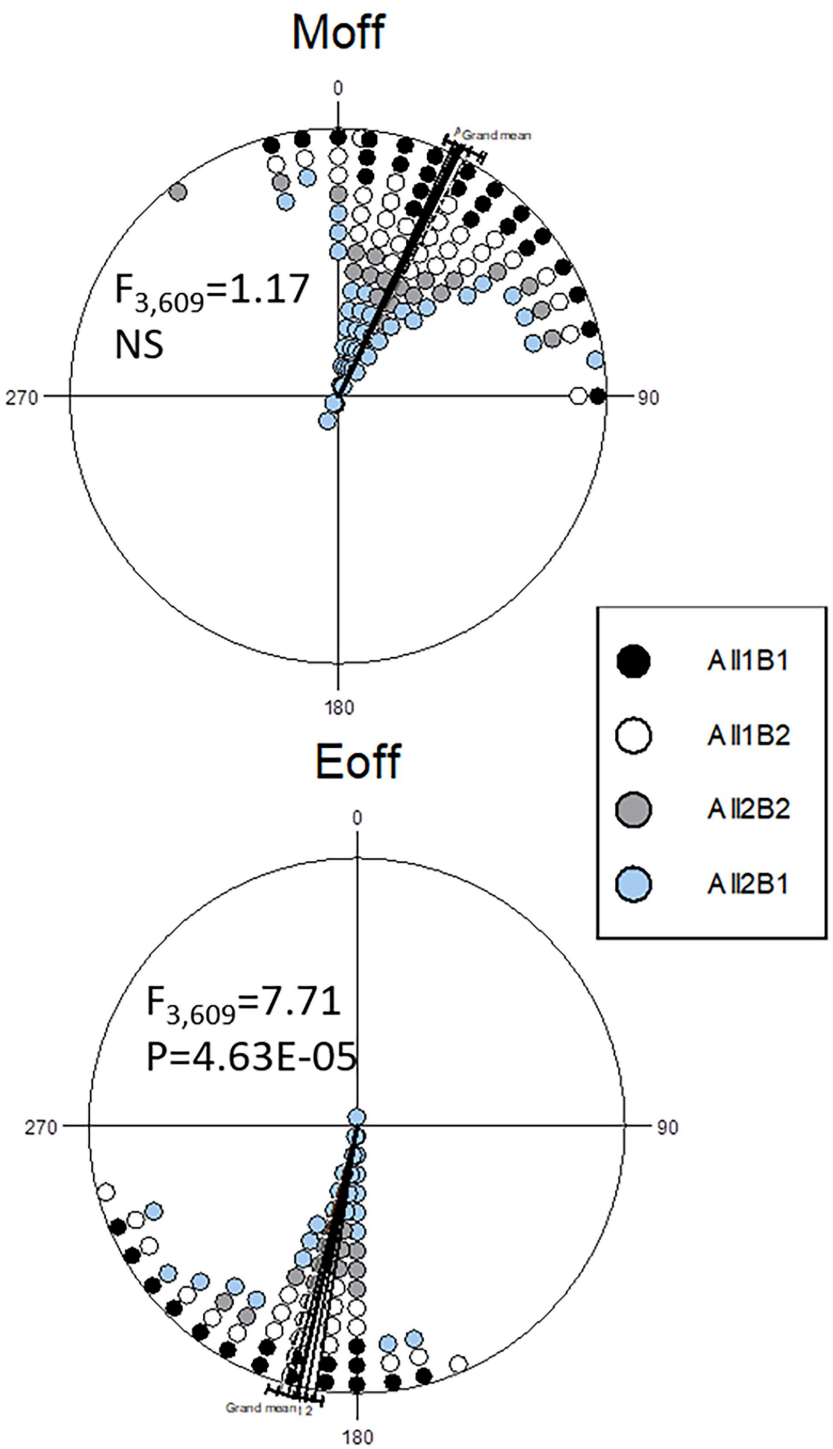
Circadian phase under light entrainment in nearly-isogenic flies carrying different *cry* haplotypes. Phase distributions of the morning (**top**) and evening (**bottom**) locomotor activity bouts are shown (offset). The sector 0°–180° represents the light phase (Zt 0–12), while dark is between 181° and 360° (Zt 12–24). Each symbol represents 7–18 observations. The circular statistics Watson-Williams *F*-test is also shown. 15° = 1 h.

Analysis of circadian phases under a light–dark cycle revealed no differences in the morning activity peak (Figure 6). In the evening peak, however, there was a small but significant difference between the haplotypes. In the NIL flies, All1B1 exhibited the most delayed evening peak, as compared to All2B2 (7.25° ~ 30 min; *F*_1,212_ = 17.47, *p* = 4.26E-05), while the smallest difference was between All1B2 and All2B1 (3.4° ~ 14 min; *F*_1,397_ = 6.05, *p* = 0.014). All1B1 was delayed, as compared to All2B1 (5.19° ~ 21 min; *F*_1,374_ = 12.34, *p* = 4.97E-04), whereas All2B2 was advanced, as compared to All1B2 (5.46° ~ 22 min; *F*_1,235_ = 10.855, *p* = 0.001; Figure 6).

**Figure 7:**
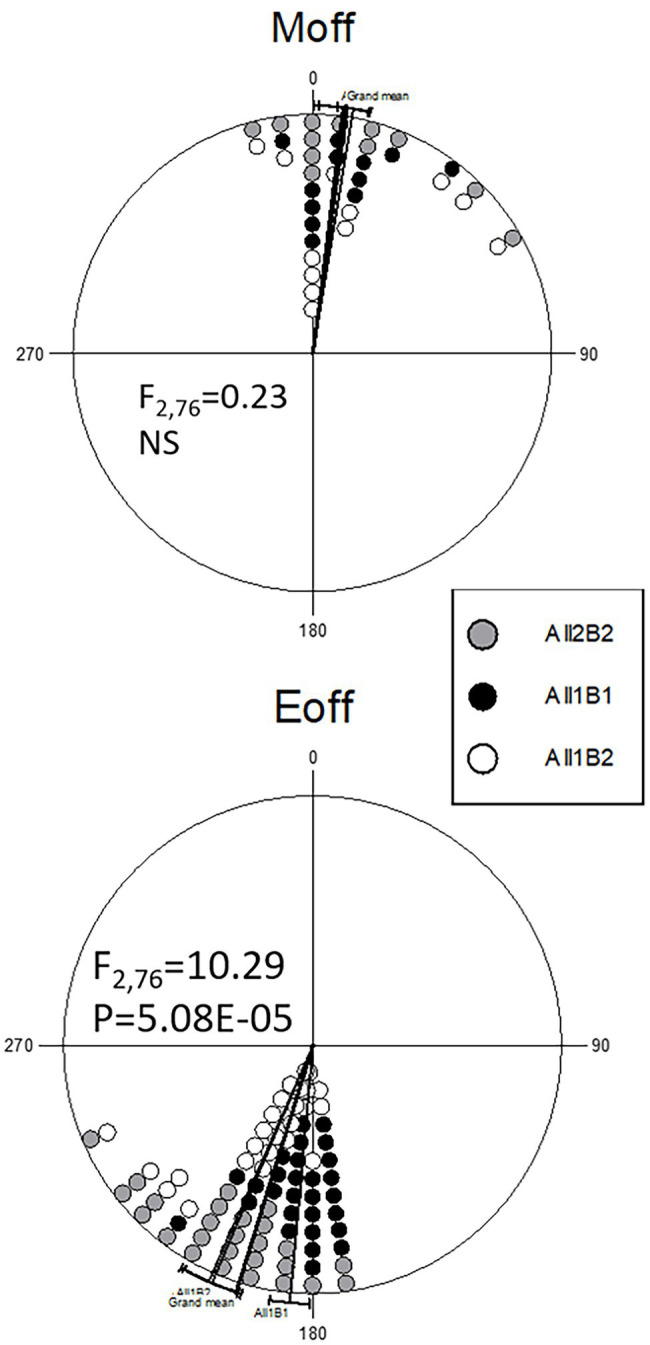
Circadian phase in transgenic flies carrying different *cry* haplotypes. Phase distributions of the morning (**top**) and evening (**bottom**) locomotor activity bouts are shown (offset). The sector 0°–180° represents the light phase (Zt 0–12), while dark is between 181° and 360° (Zt 12–24). Each symbol represents 1–3 observations. The circular statistics Watson-Williams *F*-test is also shown. 15° = 1 h.

To delineate the contributions of the All and B haplogroups to circadian behavior, we produced CRISPR-mediated transgenic strains carrying the All1B1 or All2B2 haplotypes. The *act-cas9* strain used for the transformation carried the All1B2 haplotype. We found significant differences between haplotypes in the evening phase (*F*_2,76_ = 11.29, *p* = 5.08E-05; Figure 7). The All1B1 strain was significantly more advanced than were All2B2 (18.55° ~ 74 min, *F*_1,52_ = 19.28, *p* = 5.56E-05) and the *act-cas9* All1B2 line (17.71° ~ 71 min, *F*_1,51_ = 18.04, *p* = 9.18E-05). However, there were no differences between All2B2 and the All1B2 haplotypes (*F*_1,49_ = 0.03, NS), suggesting that phase differences were due to the B haplotypes (Figure 7).

The transgenic lines DD activity phase differed significantly between haplotypes, with All1B2 being the most advanced, All1B1 most delayed, and All2B2 showing intermediate phase (Supplementary Figure S3). Interestingly, the phase differences were not due to a difference in the FRP, which did not differ between the haplotypes (*F*_2,93_ = 0.22, *p* = 0.81; Supplementary Figure S3).

Given the contribution of CRY to circadian photosensitivity (Sandrelli et al., 2007), we tested the NIL flies in light pulse experiments. We measured the phase delay after an early-night light pulse and found no differences among the haplotypes in terms of response to the light pulse (All: *F*_1,1,073_ = 0.12, NS; B: *F*_1,1,073=_0.13, NS; AllB: *F*_3,1,071_ = 1.29, NS; Supplementary Figure S1).

As *cry* affects arousal locomotor activity (Fogle et al., 2015), we also analyzed locomotor activity and sleep and associated these traits with the different haplotypes. Haplogroup All1 flies were more active than were All2 flies (*F*_1,1,141_ = 29.5, *p* = 6.84E-08), while B1 flies were more active than were B2 flies (*F*_1,1,141_ = 9.42, *p* = 0.0022; Supplementary Figure S2). All1B1 flies were more active than all other haplotypes (*F*_3,1,141_ = 24.07, *p* = 4.16E-15).

**Figure 8:**
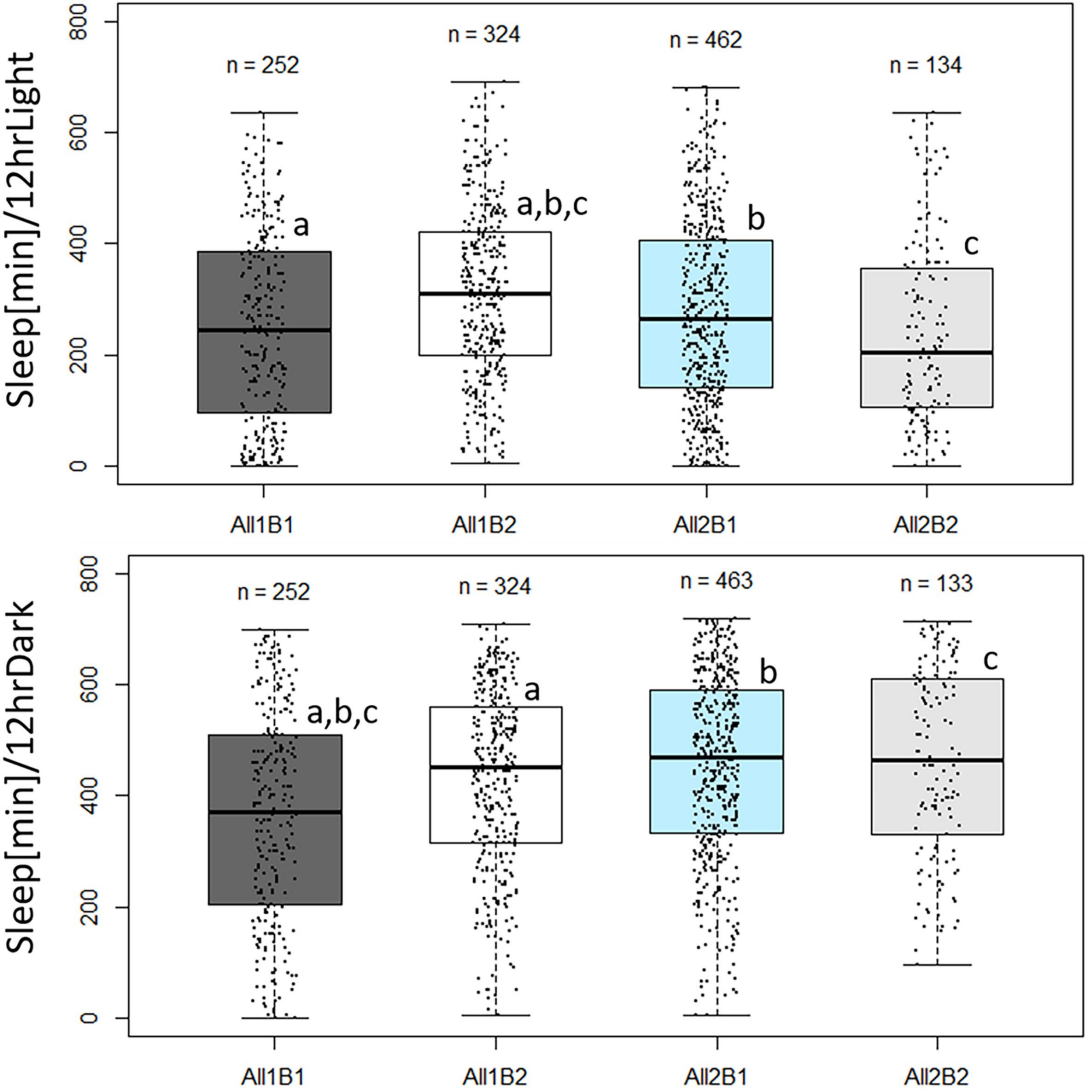
Sleep duration in *cry* haplotypes. Boxplot showing minutes of sleep during photophase (**upper panels**) and scotophase (**lower panels**). The middle line represents the median, and the box indicates the interquartile range (IQR). The whiskers signify 1.5 × IQR. The number of flies in each haplotype is indicated. Dots represent individual values. Sleep was quantified during the third day in LD. Letters indicate significant *post-hoc* comparisons (a, b, c, *p* < 0.05). Photophase sleep: All1B1 245 min ± 10.6 min (median ± SEM); All1B2 310 min ± 8.6 min; All2B1 265 min ± 7.8 min; and All2B2 205 min ± 14.0 min. Scotophase sleep: All1B1 370 min ± 12 min; All1B2 452.5 min ± 9 min; All2B1 470 min ± 7.7 min; and All2B2 465 min ± 14.5 min.

Sleep pattern also varied between the *cry* haplotypes. During daytime, All2B2 flies slept the least and All1B2 flies slept the most (*F*_3,1,139_ = 14.69, *p* = 2.19E-09; Figure 8). All1 flies slept ~35 more minutes than did All2 flies (*F*_1,1,139_ = 4.52, *p* = 0.034), while B1 flies slept ~27 min less than did B2 flies (*F*_1,1,139_ = 8.822, *p* = 0.003). Nighttime sleep also differed among haplotypes (*F*_3,1,139_ = 25.03, *p* = 1.1E-15; Figure 8) mostly due to All1B1 flies sleeping less than did the other genotypes. This is perhaps an expected result, given that All1B1 flies showed the highest amount of activity during the 24 h period (Supplementary Figure S2). All2 flies slept 45 min more than did All1 flies during nighttime, compensating for the sleep difference seen during daytime (*F*_1,1,139_ = 40.92, *p* = 2.31E-10). The B2 flies slept 15 min more during nighttime than did B1 flies, replicating the daytime difference (*F*_1,1,139_ = 5.48, *p* = 0.0194).

**Figure 9:**
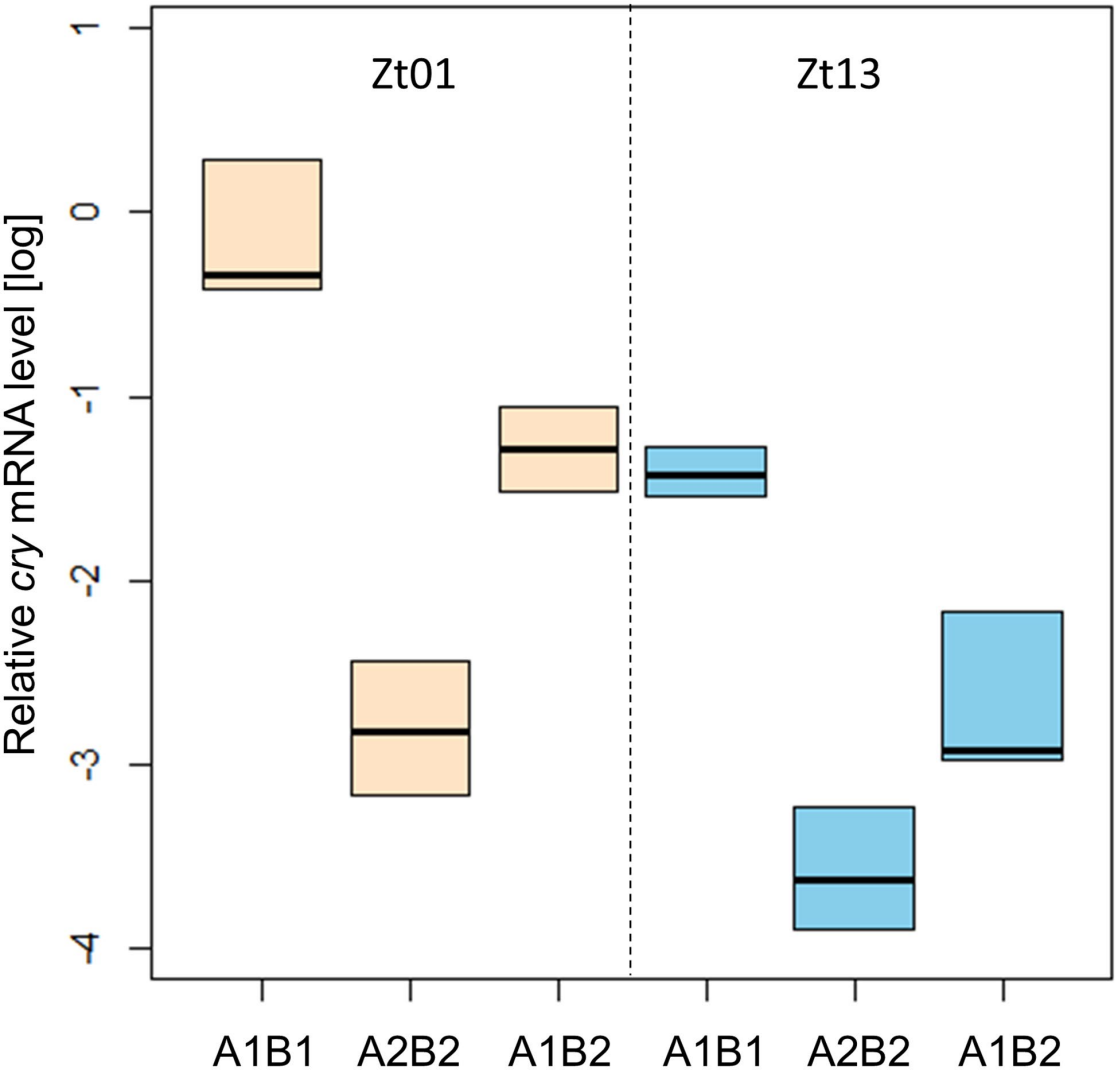
Transcriptional variation among *cry* haplotypes in transgenic flies. qPCR analysis of *cry* transcription in heads from flies sampled at Zt01 (bisque shaded) and Zt13 (blue). Boxplot represents the median and the IQR obtained from four biological replicates. The gene *Rpl32* was used as a reference gene.

We determined the transcription of *cry* under light–dark cycles at Zt01 and Zt13 by qPCR analysis (Figure 9). The abundance of *cry* mRNA differed significantly between the transgenic strains carrying the *cry-*haplotypes, at both time-points. Expression in All1B1 flies was the highest compared to the other haplotypes and was the lowest in A2B2; in All1B2 flies, expression level was intermediate (*F*_2,15_ = 35.1, *p* < 0.001).

### Geographical Distribution

**Figure 10:**
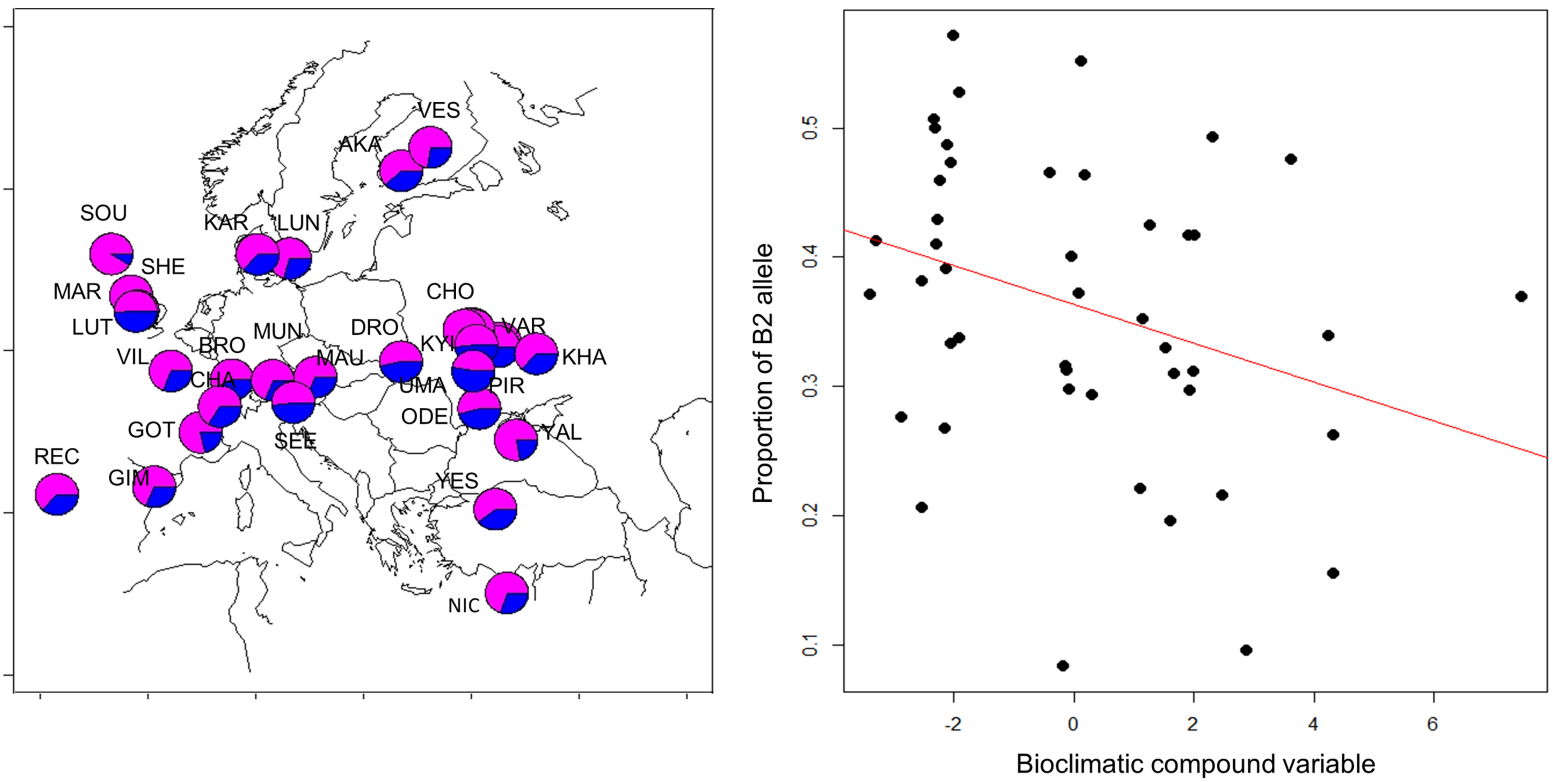
Geographical distribution of B1/B2 *cry* polymorphism. Frequency of the 12 bp indel in European populations is shown on the left (deletion: blue; insertion: magenta). Population samples were collected in 2014 and were previously described (Kapun et al., 2020). Population codes are depicted and are listed in Supplementary Table S5. Each population sample consisted of 50 flies, from which DNA was extracted, and subjected to next-generation sequencing of the pooled DNA (Kapun et al., 2020). Allele frequency in each sample was estimated by analyzing 37–139 sequencing reads spanning the B1/B2 indel. The frequency of the B2 allele (with the deletion) is highly correlated with a compound bioclimatic variable (*z* = −4.85, *p* = 1.22E-06, df = 46; see text for details).

Since geographical clines in allele frequencies are strong indicators of local adaptation, we sought to determine the spatial distribution of the B1/B2 haplotypes. We took advantage of recently published genomic sequence data collected across Europe (Kapun et al., 2020) and determined the frequency of the 12 bp indel that typifies the B1/B2 haplotypes. A generalized linear model revealed both latitude, altitude and longitude had significant, albeit small, effects on haplotype frequency. The frequency of the B2 allele (with the deletion) tended to decrease at higher latitudes (*z* = −3.182, *p* = 0.015, df = 165) and higher altitudes (*z* = −4.39, *p* = 1.14E-05) and increased toward Eastern longitudes (*z* = 5.3, 1.16E-07). To further test the signature of local adaptation caused by climatic differences, we tested association of B1/B2 polymorphism with 19 climatic variables recorded at the WorldClim database (Hijmans et al., 2005). Two compound variables, PC1 and PC2, were previously generated to sum the climatic variable set (Kapun et al., 2020). PC1 represents mostly climatic variables that distinguish warm and cold climates, while PC2 corresponds to climate seasonality. We found significant correlation of B1/B2 frequency with climatic variable PC2 (but not with PC1). The B2 allele was more frequent in climates of high “temperature seasonality standard deviation,” “Mean temperature of wettest quarter,” and “temperature annual range” (Figure 10).

## Discussion

While previous studies of molecular polymorphisms in circadian genes focused on a single variation (Costa et al., 1992; Tauber et al., 2007; Pegoraro et al., 2014), the present study aimed at simultaneous testing multiple variations across the *cry* gene. Accordingly, we generated 33 *Drosophila* nearly isogenic lines by introgressing natural *cry* alleles into the same genetic background. Using two different approaches, we identified two major haplogroups that influence circadian function. The first haplogroup (All1/2), identified by the TASSEL algorithm, forms an LD group within intron II. The second haplogroup (B1/B2) emerged from a maximum-likelihood gene tree that we constructed. This haplogroup represents a large LD cluster covering most of intron III and part of exon IV (Figures 3, 4).

Several circadian and sleep phenotypes were affected by the ALL1/2 B1/2 haplotype, including the FRP, phase (in DD and LD) and total sleep. The multiple traits with which these haplotypes are associated are consistent with *cry* being a pleiotropic gene (Damulewicz and Mazzotta, 2020). It is conceivable that these variations and other SNP across the *cry* genomic region are associated with variations in other non-circadian traits, such as geotaxis and magnetoreception. The pleiotropy of *cry* could be mediated by various molecular mechanisms, such as multiple products of the *cry* gene, different spatiotemporal patterns of expression, and different interacting proteins. A combination of these mechanisms has been shown to operate in expression of the *foraging* gene (*for*), a classic example of a behavioral pleiotropic gene (Anreiter and Sokolowski, 2019). The various *for* phenotypes, such as larval nociception, larval foraging path length, and feeding behavior of adults, are regulated by four different promoters of the gene, each dedicated to a different specific function.

The pleiotropism of *cry* may also underlie the variable pattern of polymorphism that we observed across the *cry* region. This, in turn, may reflect the compound signature of natural selection that targets different loci within the gene, each associated with a different trait. For example, Tajima’s D statistics showed both extreme positive and negative values, suggesting different selection scenarios, such as balancing and directional selection. A similar pattern was observed for *Dopa decarboxylase* (*Ddc*), another pleiotropic gene which also exhibited a variable Tajima D across its genomic region (De Luca et al., 2003). Interestingly, the excessive polymorphism in *Ddc* was associated with antagonistic pleiotropism, where traits controlled by the same gene may increase or reduce fitness under different conditions (for example, at different life stages). Antagonistic pleiotropism may increase genetic variation *via* the maintenance of intra-locus polymorphism, and lead to a balanced polymorphism (Mérot et al., 2020).

It is intriguing that circadian photosensitivity, often conceived as the canonical function of *cry* in *Drosophila*, was not associated with any genetic variation. We made a similar observation in a previous study (Pegoraro et al., 2014), where a pervasive missense SNP was associated with the FRP, circadian phase and eclosion, yet not with circadian photosensitivity. Since other light inputs contribute to light resetting of the clock (Yoshii et al., 2016), it is conceivable that evolution of this trait was mediated by genetic variations in genes other than *cry*. Indeed, QTL mapping of *Drosophila* identified a major QTL on chromosome 2 associated with circadian photosensitivity (Adewoye et al., 2017). The *tim s/ls* polymorphism was shown to be a major contributor to this QTL.

Our study suggests that from an evolutionary perspective, *cry* was the target of natural selection, largely due to its role as setting the circadian activity phase. Indeed, changes in the preferred time of activity during the day serves as a powerful adaptation of populations to local or season-specific climates and defines the ecological niche of a species (Hut et al., 2012). Previous studies have already shown that CRY co-expression with pigment-dispersing factor receptor (PDFR) in specific clock neurons is instrumental in setting the amplitude and phase of the circadian clock (Im et al., 2011). For example, testing the behavior of *cry^b^Pdf^01^* double mutants in constant darkness revealed defective rhythms that were much severe than found in each single mutant. Given the importance of the spatial expression of *cry* in the brain, it is conceivable that polymorphism in *cry* regulatory regions may lead to the generation of different subsets of neurons in which *cry* alleles are expressed, resulting in phenotypic circadian variations.

Variations in CRY expression in clock neurons may also underlie the macro-evolution of the neuronal clock network across *Drosophila* species (Helfrich-Förster et al., 2020). While Siphlodora fly species, such as *Drosophila hydei* and *Drosophila mercatorum* that reside in tropical/temperate regions show CRY (and PDF) expression like *Drosophila melanogaster*, sub-arctic species (e.g., *Drosophila ezoana* and *Drosophila littoralis*) show a distinct neural pattern; l-LNv neurons in these species do not express CRY. It is therefore plausible that adaptive molecular variations in *cry* within species populations are the foundation for species differentiation.

The geographical distribution of the B1/B2 haplotypes, specifically the 12 bp indel associated with this haplogroup, provides hints to the adaptive function of this polymorphism. Using two compound climatic variables established in a previous study (Kapun et al., 2020), we found strong correlation with PC2, a variable that reflects seasonality differences between localities. This variable strongly correlates with longitude and likely tracks the transition from an oceanic to a continental climate. The frequency of the B1/B2 polymorphism was not correlated with PC1, a climatic variable that tracks the typical temperature gradient and is highly correlated with latitude. Thus, the B1/B2 polymorphism in *cry* is likely to be driven by balancing selection that retains haplotypes that are important for seasonal adaptation and adjustment of circadian function, particularly phase of activity and sleep, to annual fluctuations of the environment. How the B1/B2 12 bp indel variation induces circadian phenotype variation has yet to be determined. An analysis of RNA binding protein motifs may hint the underlying mechanism. Using the ScanForMotifs server (Biswas and Brown, 2014), we found a reduced number of binding sites for the Elav and qkr58E-1 RNA-binding proteins in the deletion allele-containing flies. This allele also harbors a unique site for the Tis11 zinc finger protein that is not present in the insertion allele. These three proteins play roles in controlling mRNA post-transcriptional processing and catabolism (Herold et al., 2009; Choi et al., 2014; Lee et al., 2021), suggesting that B1/B2 polymorphism is associated with post-transcriptional maturation of *cry* mRNA.

## Data Availability Statement

The datasets presented in this study can be found in online repositories. The names of the repository/repositories and accession number(s) can be found at: https://www.ncbi.nlm.nih.gov/genbank/, MW758991-MW759024.

## Author Contributions

ET contributed to the conception and design of the study and wrote sections of the manuscript. MP, ES, and BF carried out the experiments. MP performed the statistical analysis and wrote the first draft of the manuscript. All authors contributed to manuscript revision, read, and approved the submitted version.

## Funding

Financial support has been provided by the Biotechnology and Biological Sciences Research Council (BBSRC, United Kingdom) grant BB/G02085X/1, which funded the postdoctoral fellowship of the lead author and supplies to conduct this research project. The Israel Science Foundation grant (1737/17) to ET also funded research supplies. The funders had no involvement in the study design, the collection, analysis and interpretation of data, the writing of the report, or in the decision to submit the article for publication.

## Conflict of Interest

The authors declare that the research was conducted in the absence of any commercial or financial relationships that could be construed as a potential conflict of interest.

## Publisher’s Note

All claims expressed in this article are solely those of the authors and do not necessarily represent those of their affiliated organizations, or those of the publisher, the editors and the reviewers. Any product that may be evaluated in this article, or claim that may be made by its manufacturer, is not guaranteed or endorsed by the publisher.
