# Supplementary material for: Nucleotide Variation in Drosophila cryptochrome Is Linked to Circadian Clock Function: An Association Analysis
Source: Front Physiol. 2022 Feb 17;13:781380. doi: 10.3389/fphys.2022.781380 (PMC8892179; doi:10.3389/fphys.2022.781380)
Supplement: Supplementary file 1 [file Table_1.DOCX]

Nucleotide variation in *Drosophila* *cryptochrome* is linked to circadian clock function: An association analysis

Mirko Pegoraro, Emily Sayegh Rezek3, Bettina Fishman and Eran Tauber

Supplementary Material

**Supplementary Table S1.** List of isofemale lines that were used to generate the NILs.

| **Name** | **Collection region** | **Year** |
| --- | --- | --- |
| MAR81_131 | Market Harborough (UK) | 2008 |
| MAR37_86 |  |  |
| MAR22_148 |  |  |
| HOJ18_110 | Hojbjeng (Denmark) | 2009 |
| HOJ12_133 |  |  |
| HOJ34_111 |  |  |
| HOJ21.1_94 |  |  |
| KOR10_12 | Korpilahti (Finland) | 2008 |
| KOR29_13 |  |  |
| KOR31_17 |  |  |
| BIT36_47 | Bitetto (Italy) | 2004 |
| REN83.31_39 | Rende (Italy) | 2006 |
| REN12.1_44 |  |  |
| REN76.2_33 |  |  |
| REN56.3_35 |  |  |
| REN52.4_37 |  |  |
| REN7.1_46 |  |  |

| **Name** | **Collect. region** | **Year** | |
| --- | --- | --- | --- |
| CORSTA2_134 | Corzes | | 2004 |
|  | st. Aegidius (Italy) | |  |
| KIL32_73 | Kilworth (UK) | | 2008 |
| KIL78_129 |  |  |  |
| KIL23_67 |  |  |  |
| KIL63_142 |  |  |  |
| KIL44_190 |  |  |  |
| KIL84_210 |  |  |  |
| KIL89_147 |  |  |  |
| KIL15_70 |  |  |  |
| KIL30_184 |  |  |  |
| STO6_21 | Stockolm (Sweden) | | 2008 |
| STO10_19 |  |  |  |
| KNO46_24 | Knoses (Creete) | | 2002 |
| BUR13_25 | Burgundy (France) | | 2000 |
| HU4.1_96 | Huten (Holland) | | 2000 |
| GOT2_125 | Goteborg (Sweden) | | 2008 |

**Supplementary Table S2.** Primers sequences

| **Primer** | **Sequence 5’-3’** |
| --- | --- |
| F-Cry3 | GGAATGGGCTAACCTACTTTTG |
| F-Cry5 | TCGACTAGAAGACGCCACCT |
| FCRY720 | TGCGTTTGGTTTTGCCCAAC |
| FCRY607 | TTTTAGCCTGCGTTTGGTTT |
| FCRY1267 | ATTTCGTTGCGCACACATAC |
| FCRY1871 | CAGGTCTCTTTCTCGCCATC |
| FCRY1964 | GGCGAGAAACGGGGATAC |
| RCRY2032 | ACGCTTTTACCCACAAAACG |
| FCRY2568 | TTCCTGGTGAGTTTAAAGCTC |
| FCRY2893 | TATCTGCTGGATGCGGATT |
| RCRY2807 | AGGTGGCGTCTTCTAGTCGA |
| RCRY3200 | AGAAGTACTCTCGCCAGATC |
| FCRY3926 | CAGCAGGAGCAGTACGAGTG |
| Rcrybgen | GGTTGCTTGAGATTGTTG |
| RCRY4784 | GCGATTAAGTCGCTGGTAGG |
| R-CRY3 | GAAGCCCATGTTGTCTCCAT |
| RCRY5293 | ATTGTAGATGGGCTGGTTGG |
| Real-time PC |  |
| cryN5 | CCGCTGACCTACCAAATGTT  cryN3   GGTGGCGTCTTCTAGTCGAG |
| cryN3 | GGTGGCGTCTTCTAGTCGAG |
| RPL32N5 | CCGCTTCAAGGGACAGTATC |
| RPL32NR | CGATCTCGCCGCAGTAAAC |

Primers sequence for sgRNA cloning in pCDF4

| 5’All1-2 | tatataggaaagatatccgggtgaacttcGtggtctcaatcaccaattggttttagagctagaaatagcaag |
| --- | --- |
| 3’All1-2 | attttaacttgctatttctagctctaaaactacacattgaagtgctccgCgacgttaaattgaaaataggtc |
| 5’B1-2 | tatataggaaagatatccgggtgaacttcGtgggcacattcatcaactcgttttagagctagaaatagcaag |
| 3’B1-2 | attttaacttgctatttctagctctaaaacgcatgtttcgctttacggCgacgttaaattgaaaataggtc |

**Supplementary Table S3.** Summary of *cry* natural genetic variation excluding 3 lines with more than 1 not sequenced polymorphic site (0.8%).

| **N of sites** | **Polymorphic sites** | **Singleton** | **Parsimony Informative sites** | **Indel**  **2-35 bp** | **Synonymous changes** | **Replacement**  **changes** |
| --- | --- | --- | --- | --- | --- | --- |
| 3848 | 123 | 54 | 69 | 20 | 32 | 16 |

**Supplementary Table S4.** Pairwise linkage Disequilibrium. Following Bonferroni correction for multiple testing, 70 comparison remain significant. Site1 and site2 indicate the genomic position of the site compares. “Dist” indicates the genomic distance in bp between sites. D, D’ and R are LD parameter calculated by DnaSP (Librado P. and Rozas, 2009 “DnaSP v5: a software for comprehensive analysis of DNA polymorphism”. *Bioinformatics*, **25**, (11), 1). FisherF is the two-tailed Fisher's exact test p value. All tests reported are significant after Bonferroni correction. The intron/exon position of the polymorphism is also reported (Intron/Exon). Nucleotide position are numbered beginning from 10 nucleotides before first exon in reference to FBgn0025680 *cry* sequence.

| **Site1** | **Site2** | **Dist** | **D** | **D'** | **R** | **Fishef** | **Intron/Exon** | **Notes** |
| --- | --- | --- | --- | --- | --- | --- | --- | --- |
| 1806 | 1814 | 8 | 0.249 | 1 | 1 | 0 | IntronII | Associate with Acrophase differences |
| 1806 | 1815 | 9 | 0.249 | 1 | 1 | 0 | IntronII | Associate with Acrophase differences |
| 1806 | 1816 | 10 | 0.249 | 1 | 1 | 0 | IntronII | Associate with Acrophase differences |
| 1806 | 1828 | 22 | 0.213 | 1 | 0.873 | 0 | IntronII | Associate with Acrophase differences |
| 1814 | 1815 | 1 | 0.249 | 1 | 1 | 0 | IntronII | Associate with Acrophase differences |
| 1814 | 1816 | 2 | 0.249 | 1 | 1 | 0 | IntronII | Associate with Acrophase differences |
| 1814 | 1828 | 14 | 0.213 | 1 | 0.873 | 0 | IntronII | Associate with Acrophase differences |
| 1815 | 1816 | 1 | 0.249 | 1 | 1 | 0 | IntronII | Associate with Acrophase differences |
| 1815 | 1828 | 13 | 0.213 | 1 | 0.873 | 0 | IntronII | Associate with Acrophase differences |
| 1816 | 1828 | 12 | 0.213 | 1 | 0.873 | 0 | IntronII | Associate with Acrophase differences |
| 1828 | 1883 | 54 | 0.193 | 0.853 | 0.796 | 0 | IntronII | Associate with Acrophase differences |
| 1883 | 1896 | 13 | 0.198 | 0.856 | 0.8 | 0 | IntronII | Associate with Acrophase differences |
| 1883 | 1916 | 33 | 0.198 | 0.856 | 0.8 | 0 | IntronII | Associate with Acrophase differences |
| 1883 | 1945 | 61 | 0.227 | 1 | 0.934 | 0 | IntronII | Associate with Acrophase differences |
| 1896 | 1916 | 20 | 0.249 | 1 | 1 | 0 | IntronII |  |
| 1896 | 1945 | 48 | 0.213 | 1 | 0.873 | 0 | IntronII |  |
| 1916 | 1945 | 28 | 0.213 | 1 | 0.873 | 0 | IntronII |  |
| 2432 | 2480 | 48 | 0.211 | 1 | 0.929 | 0 | ExonIII | 2480 is E335DD |
| 2432 | 2516 | 84 | 0.189 | 0.85 | 0.85 | 0 | ExonIII |  |
| 2432 | 2517 | 85 | 0.189 | 0.85 | 0.85 | 0 | ExonIII | 2517 is D348N |
| 2480 | 2516 | 36 | 0.211 | 1 | 0.929 | 0 | ExonIII | 2480 is E335DD |
| 2480 | 2517 | 37 | 0.211 | 1 | 0.929 | 0 | ExonIII | 2480 is E335DD |
| 2516 | 2517 | 1 | 0.222 | 1 | 1 | 0 | ExonIII | 2517 is D348N |
| 2843 | 2852 | 9 | 0.24 | 1 | 1 | 0 | ExonIII |  |
| 2843 | 2890 | 47 | 0.193 | 0.853 | 0.796 | 0 | ExonIII | 2890 in IntronIII |
| 2843 | 2912 | 69 | 0.193 | 0.853 | 0.796 | 0 | ExonIII | 2912 in IntronIII |
| 2843 | 2916 | 73 | 0.193 | 0.853 | 0.796 | 0 | ExonIII | 2916 in IntronIII |
| 2852 | 2890 | 38 | 0.193 | 0.853 | 0.796 | 0 | ExonIII | 2890 in IntronIII |
| 2852 | 2912 | 60 | 0.193 | 0.853 | 0.796 | 0 | ExonIII | 2912 in IntronIII |
| 2852 | 2916 | 64 | 0.193 | 0.853 | 0.796 | 0 | ExonIII | 2916 in IntronIII |
| 2890 | 2912 | 22 | 0.246 | 1 | 1 | 0 | IntronIII |  |
| 2890 | 2916 | 26 | 0.246 | 1 | 1 | 0 | IntronIII |  |
| 2912 | 2916 | 4 | 0.246 | 1 | 1 | 0 | IntronIII |  |
| 2964 | 3026 | 62 | -0.202 | -1 | -0.818 | 0 | IntronIII | Ancestral Reconstruction |
| 2964 | 3027 | 63 | -0.202 | -1 | -0.818 | 0 | IntronIII | Ancestral Reconstruction |
| 2964 | 3075 | 104 | 0.246 | 1 | 1 | 0 | IntronIII | Ancestral Reconstruction |
| 2964 | 3106 | 130 | 0.246 | 1 | 1 | 0 | IntronIII | Ancestral Reconstruction |
| 2964 | 3107 | 131 | 0.231 | 1 | 0.935 | 0 | IntronIII | Ancestral Reconstruction |
| 2964 | 3115 | 139 | 0.246 | 1 | 1 | 0 | IntronIII | Ancestral Reconstruction |
| 2964 | 3120 | 144 | 0.246 | 1 | 1 | 0 | IntronIII | Ancestral Reconstruction |
| 2975 | 2979 | 4 | 0.232 | 1 | 1 | 0 | IntronIII |  |
| 2975 | 2984 | 9 | 0.232 | 1 | 1 | 0 | IntronIII |  |
| 2975 | 3005 | 30 | 0.232 | 1 | 1 | 0 | IntronIII |  |
| 2979 | 2984 | 5 | 0.232 | 1 | 1 | 0 | IntronIII |  |
| 2979 | 3005 | 26 | 0.232 | 1 | 1 | 0 | IntronIII |  |
| 2984 | 3005 | 21 | 0.232 | 1 | 1 | 0 | IntronIII |  |
| 3026 | 3027 | 1 | 0.249 | 1 | 1 | 0 | IntronIII | Ancestral Reconstruction |
| 3026 | 3075 | 42 | -0.202 | -1 | -0.818 | 0 | IntronIII | Ancestral Reconstruction |
| 3026 | 3106 | 68 | -0.202 | -1 | -0.818 | 0 | IntronIII | Ancestral Reconstruction |
| 3026 | 3115 | 77 | -0.202 | -1 | -0.818 | 0 | IntronIII | Ancestral Reconstruction |
| 3026 | 3120 | 82 | -0.202 | -1 | -0.818 | 0 | IntronIII | Ancestral Reconstruction |
| 3026 | 3209 | 171 | -0.218 | -1 | -0.875 | 0 | IntronIII | Ancestral Reconstruction |
| 3027 | 3075 | 41 | -0.202 | -1 | -0.818 | 0 | IntronIII | Ancestral Reconstruction |
| 3027 | 3106 | 67 | -0.202 | -1 | -0.818 | 0 | IntronIII | Ancestral Reconstruction |
| 3027 | 3115 | 76 | -0.202 | -1 | -0.818 | 0 | IntronIII | Ancestral Reconstruction |
| 3027 | 3120 | 81 | -0.202 | -1 | -0.818 | 0 | IntronIII | Ancestral Reconstruction |
| 3027 | 3209 | 170 | -0.218 | -1 | -0.875 | 0 | IntronIII | 3209 in Exon IV |
| 3075 | 3106 | 26 | 0.246 | 1 | 1 | 0 | IntronIII | Ancestral Reconstruction |
| 3075 | 3107 | 27 | 0.231 | 1 | 0.935 | 0 | IntronIII | Ancestral Reconstruction |
| 3075 | 3115 | 35 | 0.246 | 1 | 1 | 0 | IntronIII | Ancestral Reconstruction |
| 3075 | 3120 | 40 | 0.246 | 1 | 1 | 0 | IntronIII | Ancestral Reconstruction |
| 3106 | 3107 | 1 | 0.231 | 1 | 0.935 | 0 | IntronIII | Ancestral Reconstruction |
| 3106 | 3115 | 9 | 0.246 | 1 | 1 | 0 | IntronIII | Ancestral Reconstruction |
| 3106 | 3120 | 14 | 0.246 | 1 | 1 | 0 | IntronIII | Ancestral Reconstruction |
| 3107 | 3115 | 8 | 0.231 | 1 | 0.935 | 0 | IntronIII | Ancestral Reconstruction |
| 3107 | 3120 | 13 | 0.231 | 1 | 0.935 | 0 | IntronIII | Ancestral Reconstruction |
| 3115 | 3120 | 5 | 0.246 | 1 | 1 | 0 | IntronIII | Ancestral Reconstruction |
| 3120 |  |  |  |  |  |  | IntronIII | Ancestral Reconstruction |
| 3209 |  |  |  |  |  |  | ExonIV | Ancestral Reconstruction |
| 3230 | 3239 | 9 | 0.21 | 1 | 1 | 0 | ExonIV |  |
| 3230 | 3257 | 27 | 0.21 | 1 | 1 | 0 | ExonIV |  |
| 3239 | 3257 | 18 | 0.21 | 1 | 1 | 0 | ExonIV |  |

**Supplementary Table S5.** Population names and abbreviations. Populations are from (Kapun et al 2020, *Mol. Biol. Evol*. **37**, 2661–2678.).

| **Location** | **Code** | **Country** |  |
| --- | --- | --- | --- |
| Mauternbach | MAU | Austria |  |
| Yesiloz | YES | Turkey |  |
| Viltain | VIL | France |  |
| Gotheron | GOT | France |  |
| Sheffield | SHE | UK |  |
| South Queensferry | SOU | UK |  |
| Nicosia | NIC | Cyprus |  |
| Market Harborough | MAR | UK |  |
| Lutterworth | LUT | UK |  |
| Broggingen | BRO | Germany |  |
| Yalta | YAL | Ukraine |  |
| Odesa | ODE | Ukraine |  |
| Kyiv | KYI | Ukraine |  |
| Varva | VAR | Ukraine |  |
| Piryuatin | PIR | Ukraine |  |
|  |  |  |  |
| **Location** | **Code** | **Country** |  |
| Drogobych | DRO | Ukraine |  |
| Chornobyl Yaniv | CHO | Ukraine |  |
| Lund | LUN | Sweden |  |
| Munich | MUN | Germany |  |
| Recarei | REC | Portugal |  |
| Gimenells | GIM | Spain |  |
| Akaa | AKA | Finland |  |
| Vesanto | VES | Finland |  |
| Karensminde | KAR | Denmark |  |
| Chalet Gobet | CHA | Switzerland |  |
| Seeboden | SEE | Austria | |
| Kharkiv | KHA | Ukraine | |
| Chornobyl Applegarden | CHO | Ukraine | |
| Kyiv | KYI | Ukraine | |
| Uman | UMA | Ukraine | |
|  |  |  | |


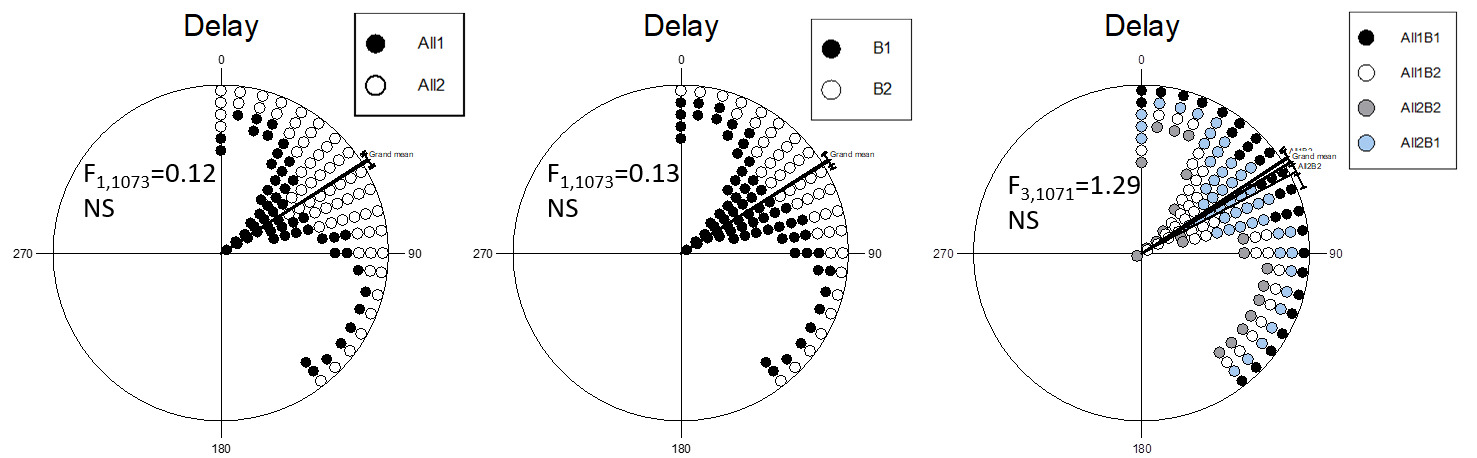


**Supplementary** **Figure S1**. Phase response (delay) to light pulse at ZT15. **Left** All1 (57.33˚± 30.25˚, Mean vector ± circular standard deviation, n=512) and All2 (57.97˚ ± 29.10˚, n=563) delay. **Middle** B1 (57.41˚ ± 30.14˚, n=665) and B2 (58.07˚ ± 28.84˚, n=410) delay. **Right** All1B1 (58.58˚ ± 34.10˚, n=222), All1B2 (56.44˚ ± 27.01˚, n=290), Al2B1 (58.86˚ ± 27.99˚, n=443), All2B2 (62.24˚ ± 32.56˚, n=120) delay. Each Symbol=11 observations. 15˚=1hour.

**
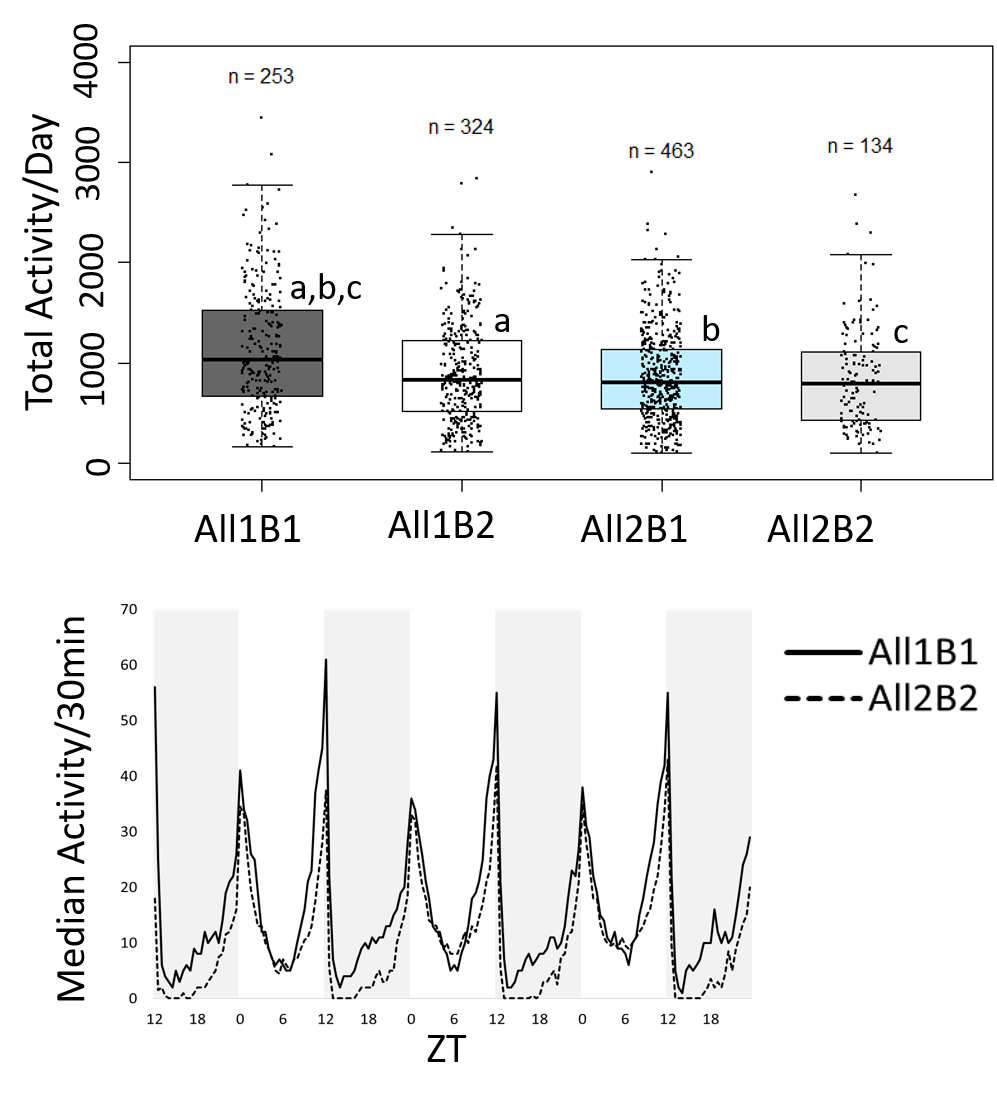
 Supplementary Figure S2.** Comparison of Total activity in *cry* haplotypes. Boxplot showing Total activity on the 3rd day in LD (upper panel) for All1B1 (1031 ± 39.15), All1B2 (825.5 ± 26.95), All2B1 (808 ± 21.04) and All2B2 (798 ± 42.17) flies. Letters indicate post doc significant comparisons (a, b and c: p<0.001). The boxplot middle line represents the median, and the box indicated the interquartile range (IQR). The whiskers signify the 1.5 times the IQR. Number of flies in each haplotype is indicated. Dots are individual values. Activity profiles (bottom panel) of All1B1 (n=275) and All2B2 (n=148) flies in LD at 25˚C. Shaded areas represent nights (light off).


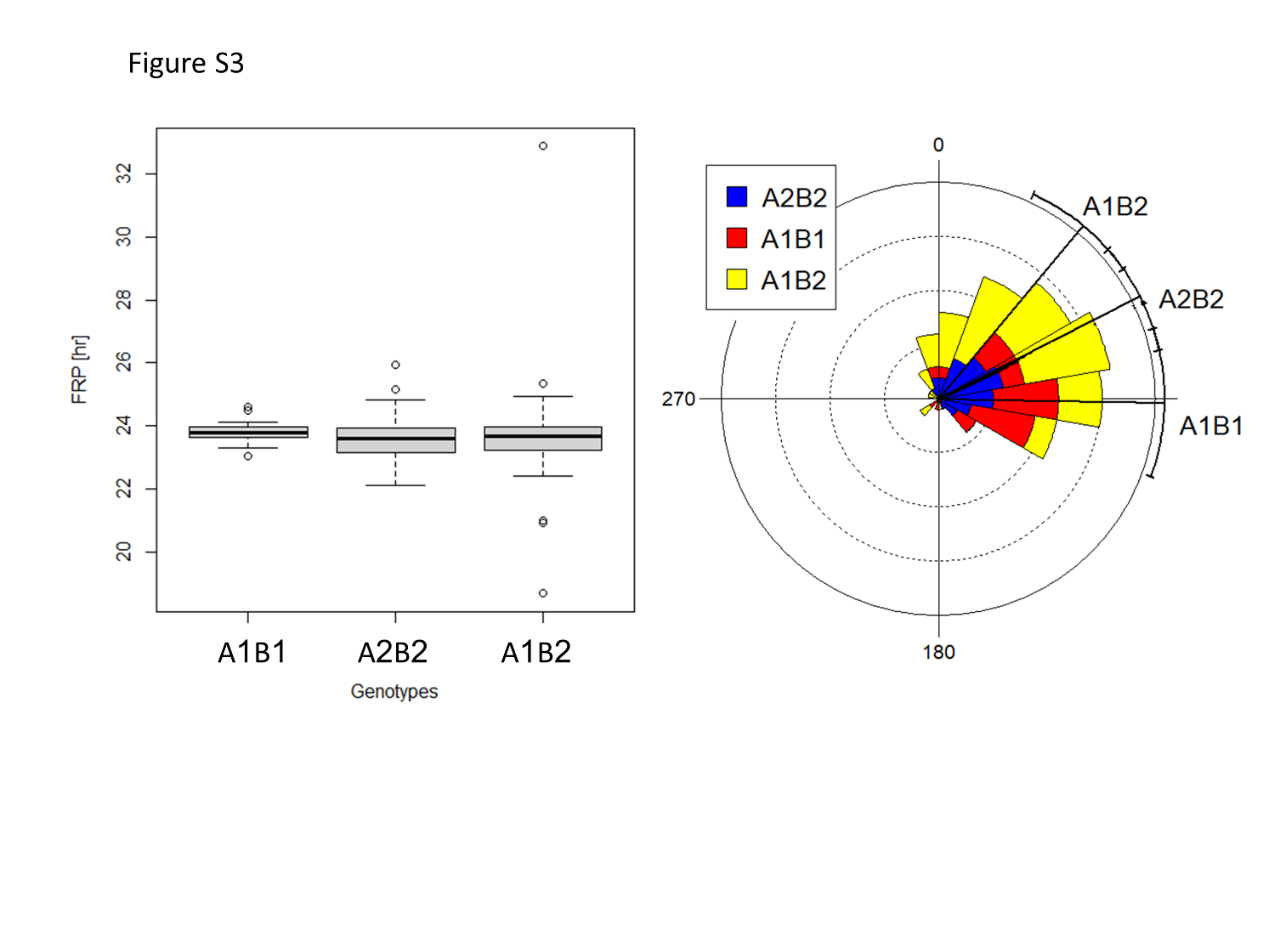


**Supplementary Figure S3.** Locomotor activity of *cry* haplotypes under free-running conditions. The FRP of All1B1 (n=23), All1B2 (n=31) and All1B2 (n=42) transgenic flies is depicted on the left (one-way ANOVA, F_2,93_ = 0.22, p =0.81, power(f=.4)=.93). The circular plot (right panel) shows the acrophase histogram of the different haplotypes (Watson-William F test, F_2,93_= 8.45, p < 0.001). The lines are the mean vectors and their direction indicates the mean phase (±95% confidence intervals). The sector 0–180° represents the subjective day (Ct 0–12), while subjective night is between 181–360° (Ct 12–24).
